# Tunable Surface Plasmon and Phonon Polariton Interactions for Moderately Doped Semiconductor Surfaces

**DOI:** 10.1038/srep34071

**Published:** 2016-10-04

**Authors:** Mohsen Janipour, Ibrahim Burc Misirlioglu, Kursat Sendur

**Affiliations:** 1 Faculty of Engineering and Natural Science, Sabancı University, Orhanlı – Tuzla, Istanbul, Turkey; 2 Department of Electrical Engineering, Iran University of Science and Technology, Tehran, Iran

## Abstract

Spatial charge distribution for biased semiconductors fundamentally differs from metals since they can allow inhomogeneous charge distributions due to penetration of the electric field, as observed in the classical Schottky junctions. Similarly, the electrostatics of the dielectric/semiconductor interface can lead to a carrier depletion or accumulation in the semiconductor side when under applied bias. In this study, we demonstrate that the inhomogeneous carrier accumulation in a moderately *p*-doped GaAs–dielectric interface can be tailored for tunable plasmonics by an external voltage. Solving Maxwell’s equations in the doped GaAs-dielectric stack, we investigate the tunability of the surface plasmon and phonon polaritons’ interaction via an external bias. The plasmonic mode analysis of such an interface reveals interesting dispersion curves for surface plasmon and phonon polariton interactions that are not possible in metals. We show that the plasmon dispersion curve can be engineered through an external bias using the inherent properties of the *p*-doped GaAs– dielectric interface.

The conversion of light to propagating oscillations of free electrons at a metal-dielectric interface, namely plasmonics, has become an important field of study for applications ranging from the new generation of electronics to solar energy harnessing and sensing. It is well known that at the interface of a noble metal and a dielectric (DE) medium, surface plasmon polaritons (SPPs) can be excited under special conditions^1^,^2^, which are due to the resonant coupling of the incident electromagnetic field and free electrons of the metal medium in the optical regime^3^,^4^,^5^. Nevertheless, this optical behavior depends on plasma frequency, which is a strong function of the density of free carriers of the metallic medium. For lower frequencies, such as the terahertz regime, this phenomenon is more difficult to achieve with metals. Owing to their intrinsic properties, metals act as perfect electrical conductors in terahertz and lower frequencies^6^,^7^,^8^, thus a metal-dielectric interface configuration can have challenges for tunable far-infrared (IR) plasmonic applications. Various techniques other than the classical metal-dielectric interfaces have been used to excite SPPs at lower frequency bands such as IR and lower. For example, Babuty *et al*. showed that by periodically patterning the metallic contact of a mid-IR quantum cascade laser, the surface plasmons (SPs) can propagate at the metal-semiconductor (SC) boundary^9^. Li *et al*. demonstrated theoretically and experimentally a semiconductor plasmonic terahertz waveguide which can be obtained using a doped silicon medium as a conductive substrate^10^. Further building on these investigations^10^,^11^, Ghosh *et al*. showed that Ge/Si combination can be used as a SP sensor with the application of photo detections^12^. The main focus of these works is the use of a semiconducting medium to excite surface plasmons, which is attractive for longer wavelengths than typical metal-dielectric interfaces. For instance, Law *et al*. recently presented a plasmonic nanoantenna for mid-IR sensing using a heavily-doped InAs^13^. That work also demonstrated that in doped SCs, the resonances due to free carriers can be modeled by Drude formalism plus a Lorentz oscillator approach for phonon resonances^14^,^15^,^16^ which may excite surface phonon polaritons (SPhPs). These studies explored the optical regime of far-IR frequencies for plasmonic device design and shed light on the physics of the problem^17^,^18^. In essence, SPhPs are due to the interaction of polar optical phonons with long-wavelength incident electromagnetic fields which can occur in SiC^18^,^19^,^20^, hexagonal BN^21^,^22^,^23^,^24^, GaAs^25^,^26^, InP^27^, and CaF_2_^28^.

In this work, our aim is to demonstrate that the charge distribution at a moderately doped DE–SC interface can be tailored by an external voltage, which leads to position dependency of a carrier density function that can exhibit a highly unconventional plasmonic response to incoming excitation. This study is a theoretical and numerical one based on the analyses of plasmon and phonon-polariton modes at the DE-SC interface. We note that the control of the carrier density and distribution in the semiconductor theoretically allows control of the above mentioned resonances. We demonstrate that the resonances can be tuned by controlling the carrier density at the DE-SC interface via an external bias. We hope that our results will lead to a better understanding and interpretation of the outcomes of previous works on similar systems as well identify new areas of study on resonance frequency control at DE-SC thin film stacks. Under such inhomogeneous carrier distributions near a DE-SC interface (that can be controlled via an externally applied voltage), we derived the dispersion relations for plasmons and phonon interactions, providing the relevant plots for GaAs with *p*-type doping (*p*GaAs). In this manuscript, we obtained the results for a p-type GaAs as the SC material, however, the same concept can be extended to other SC material systems as well. We chose *p*GaAs as a model system since there is abundant experimental data on *p*GaAs and its semiconducting parameters (such as carrier mobility, dielectric relaxation time, and carrier relaxation time for various doping levels) are well defined^14^,^29^,^30^. Solving the Maxwell’s equations for a *p*GaAs interfacing a dielectric, we demonstrate the voltage controlled tunability of plasmon and phonon polaritons. We show that appropriately doped SC slabs interfacing a dielectric medium can be tailored to control SPP/SPhP via an external bias and that this is strongly coupled to carrier density at the interface.

## Charge Distribution at a Moderately *p*GaAs-Dielectric Interface

Spatial charge distribution in a doped SC medium with a bandgap close to that of Si fundamentally differs from metals due to larger skin depths and, as the electric field can penetrate into the SC, inhomogeneous spatial charge distribution can exist under an applied potential. A good example of inhomogeneous charge distribution is the classical Schottky junction. In a Schottky junction, the carriers are redistributed due to the difference between the Fermi levels of the SC and the metal contact. This can generate a depletion zone in the SC side which decays either gradually or abruptly into the bulk and corresponds to a thermodynamic equilibrium state. The distance encompassing the decay of the ionized dopants is known as the Debye screening length. While such descriptions are well established^15^, the tailoring of carrier distribution in an SC in contact with a metal or a dielectric forms the basis of today’s modern electronics and is important for plasmonic device considerations. Among SC materials, GaAs has been getting attention for plasmonic applications^31^ and is the SC for which we obtain the carrier distribution and analyze its SPP and SPhP characteristics in this work. We expect that the carrier densities in a moderately *p*-doped (10^17–18^ cm^−3^) GaAs would be 4–5 orders of magnitude less than in a good metal. Furthermore, due to strong dependency of the plasma frequency on the carrier density, it is expected that the SPP propagations at the doped GaAs interfaces may occur at lower frequencies such as the IR frequency band^31^. It has been shown that doped SCs such as SiC particles are capable of supporting surface waves in the 10–12.5 *μm* range^17^. In addition, wide bandgap materials systems such as III-Nitrides have been shown to support plasmons in the 11–18 *μm* range^21^,^22^, and ZnO can do so in the near-IR regime^32^. Moreover, VO_2_, a material getting attention for the insulator-to-metal transition in the vicinity of room temperature, has been demonstrated to allow induction of near-IR plasmons in submicron film form (Kretschmann configuration) via external thermal control^33^. Therefore, enabling plasmon oscillations even in wide bandgap SCs with impurities is a possibility as the above works reveal, but it is another question whether these plasmons can be frequency tuned. Due to the finite penetration of the electric field in these materials, the latter should be possible as we show for *p*GaAs in the coming sections. In another interesting case, Misirlioglu *et al*. showed that the carrier density at the SC-ferroelectric interface can be varied orders of magnitude through polarization of the ferroelectric layer, which is a polar dielectric capable of generating very strong fields near the interface^34^.

Thus, in analogy with the optical regime SPPs, one has to consider the carrier distribution at a DE-*p*GaAs interface for plasmonic applications. With this in mind, we consider a bias on the DE-SC interface that can be controlled externally. We used lateral boundary conditions to ensure that we have a homogeneous voltage distribution across the stack. Moreover, from a practical point of view, to avoid edge effects that might interfere with the resonances reported here, the lateral dimensions of a device should be, in theory, orders of magnitude greater than the wavelength of the excitation. Although in the theoretical calculations the interface of semi-infinite SC and DE mediums is considered, the thickness of these regions should be reasonable to allow the carrier density control within a realistic range of the bias voltages (a few volts or more). For studying the plasmonic effects at such interfaces one needs to find the carrier accumulation inside the SC region and also near the interface which can be position-dependent due to the applied bias. The localized spatial carrier density in the *p*GaAs region can be obtained using Poisson’s equation:


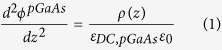


where *ε*_*DC*,*pGaAs*_ is the relative static dielectric constant of the doped GaAs medium, *ε*_0_ is the permittivity of vacuum, and *ρ*(*z*) represents the density of free carriers, respectively. It should be noted that in the DE medium *ρ* = 0 which leads to the solution of the Laplace’s equation in this region:


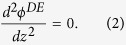


The spatial distribution of the carriers in each region can be obtained by applying the continuity condition of *ϕ*^*pGaAs*^ = *ϕ*^*DE*^ at the interface (i.e. *z* = 0). As we control the bias on the semiconductor side, a negative bias depletes the holes (the type of carriers in this work) at the dielectric/semiconductor interface where the plasmon resonances are expected to occur. Therefore, a negative bias was not pursued at any stage of the study. In order to achieve the carrier distribution at the junction, in equation (1), it is considered that 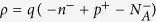 with:


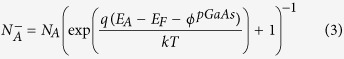



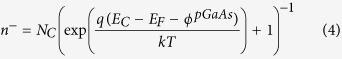



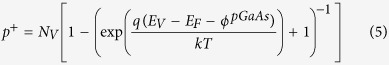


where 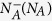 is the ionized (total) acceptor density for the *p*GaAs, *n*^−^ is the electron density, *p*^+^ is the hole density, *N*_*C*_ is the effective density of states at the bottom of the conduction band, *N*_*V*_ is the effective density of states at the top of the valence band, *E*_*C*_ is the energy of an electron at the bottom of the conduction band, *E*_*V*_ is the energy of an electron at the top of the valence band, *E*_*F*_ is the Fermi level, *ϕ*^*pGaAs*^ is the local electrostatic potential and *E*_*A*_ is the ionization energy of the acceptor atom which is taken according to the top of the valence band. In order to perform the calculations including the band bending in the SC medium, it is essential to know *E*_*F*_ of the *p*GaAs as a function of dopant concentration which can be calculated from the charge neutrality condition:


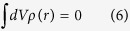


Coming into contact with a metal electrode that is expected to have a much higher population density near the Fermi level, the *p*GaAs will equilibrate with the Fermi level of the metal via carrier transfer at the interface. Due to the discontinuity of the dielectric constants at the DE*-p*GaAs junction, a local electric field and therefore a charge accumulation whose magnitude will depend on voltage and the Fermi level of the system can be anticipated. For the *p*-doped GaAs, depending on the bias on the top metal electrode, there would be either exposed ionized acceptors or free carriers (holes in the SC region) near the interface that forms the basis of the analysis carried out in this work.

Relatively low carrier density in the SC region allows the penetration of the bias field into the *p*GaAs medium, which causes inhomogeneous charge distributions on the SC side, especially near the interface. As we demonstrate later, this inhomogeneous carrier distribution near the interface leads to rather interesting optical resonances in the far-IR frequencies. Away from the interface the homogeneous charge distribution, i.e., 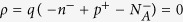 condition would be restored in the bulk for a sufficiently thick SC medium. In this work, due to the finiteness of the GaAs thickness, we do not get a region where *ρ* = 0 unless very high dopant concentrations are considered (small screening lengths similar to metallic behavior), which are both outside the scope of the work in addition to converging to a DE-metal case that works at optical frequencies. We provide carrier density distribution results for moderate doping level obtained by solving equations (1), (2) and equations (3), (4).

**Figure 1:**
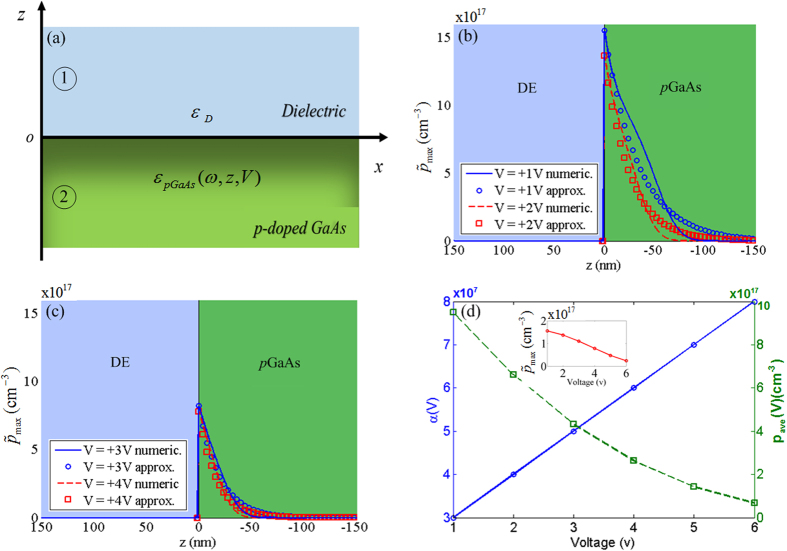
(**a**) Description of the considered *p*GaAs, *ε*_*pGaAs*_(*ω*, *z*, *V*), interfacing a dielectric medium with *ε*_*D*_. Maximum carrier density plots obtained from the numerical solution of equation (1) and equations (3)–(5), 21, 21 for (**b**) V = +1 V (solid-line), and +2 V (dashed-line) bias, (**c**) V = +3 V (solid-line), and +4 V (dashed-line) bias. Approximate fit using a form 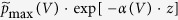 is also given in all plots; [(**b**) +1 V (circles), and +2 V (cubes)], [(**c**) +3 V (circles), and +4 V (cubes)]; where 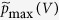 is the voltage dependent maximum carrier density at the interface. (**d**) The average carrier density [i.e.*p*_*ave*_(*V*)]; (dashed-line) as a function of bias and the plot of spatial decay coefficient [i.e. *α*(*V*)] along z-axis (solid-line) obtained from the approximation of the numerical data for the applied bias voltages of interest in this work. The inset curve shows the density of maximum free carrier density occurring at the interface versus the bias voltage.

Figure 1a depicts the schematic representation of the considered DE-*p*GaAs interface with individual *ε*_*pGaAs*_(*ω*, *z*, *V*) and *ε*_*D*_ dielectric permittivities, respectively. The maximum carrier density distribution [i.e., 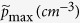] achieved from numerical calculations of equation (1) and equations (3, 4, 5) for Fig. 1b, V = +1 V (solid-line), and +2 V (dashed-line); Fig. 1c, V = +3 V (solid-line), and +4 V (dashed-line). A curve fit approximation to the numercial data using 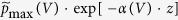 is also shown in the plots and *α* is a decay coefficient that is used to approximate the numerical solution [Fig. 1b +1 V (circles), and +2 V (cubes)]; [Fig. 1c +3 V (circles), and +4 V (cubes)]. Figure 1d represents the average density of free carriers (cubes); [i.e. *p*_*ave*_(*V*)], and spatial decay rate (circles); [i.e. *α*(*V*)] versus changing the bias voltage. The inset shows the voltage variations of the maximum value of free carrier density [i.e. 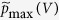]. In addition, since the presented study consists of static solution of the carrier distribution at the interface which strongly affects the solution of the wave equation in the doped GaAs region as an inhomogeneous medium, we supposed both the relative static dielectric constant (i.e. *ε*_*DC*,*pGaAs*_), and also the dielectric constant of the GaAs medium at high frequencies; (i.e. *ε*_∞,*pGaAs*_) in corresponding solutions, respectively. The data parameters used to solve equations (1, 2, 3, 4, 5) and also to calculate the dielectric permittivity function of the *p*GaAs medium can be found in Tables 1 and 2 14,35,36.

The main effect of the doping is to increase the volumetric density of carriers relative to an intrinsic GaAs medium and render plasmonic effects possible. We assume that the amount of doping here does not alter the symmetry or structure of the GaAs medium and only affects its dielectric function. One would expect that, under bias, there will be a jump in dielectric displacement at the DE-*p*GaAs interface, causing carrier accumulation (holes for *p*GaAs) at or near the interface. This is indeed the case as shown in Fig. 1 and the carrier accumulation is a function of bias on the SC side. The electrostatic potential inside the doped GaAs medium is rather inhomogeneous due to the presence of space charges (carriers and ionized acceptors) and this results in an almost exponentially decaying carrier density away from the DE-*p*GaAs interface. Increasing the positive bias voltage on the GaAs side leads to a decrease in hole densities which is also energetically more favorable for the system. The band edges also move with respect to the Fermi level under bias. The results demonstrated in Fig. 1b,c suggest that the average and maximum density of the carriers can be tuned significantly by changing the applied DC voltage on the doped GaAs side. Under such conditions, the dielectric function of the *p*GaAs medium may be modeled with a classical Drude-Lorentz model^14^,^15^, where the Drude and Lorentz parts are correspondingly used to incorporate the contribution of free carriers (voltage dependent) and lattice phonon resonances (approximately voltage independent)^29^,^30^. In contrast to the well-known noble metals in which the free electrons are homogeneously distributed in the metallic medium, the average density of carriers, as shown in Fig. 1d, takes an almost exponentially variation in a moderately *p-*doped GaAs region. Thus, the dielectric function of the *p*GaAs side which can behave analogus to a spatial inhomogeneous medium may be presented as:


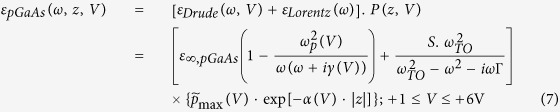


where *ω*_*p*_(*V*) and *γ*(*V*) are the voltage tunable plasma frequency and collision rate of free carriers, *ε*_∞,*pGaAs*_ is the dielectric constant of the doped GaAs at high-frequencies, *ω*_*TO*_ and Γ are the transverse optical (TO) phonon resonance frequency and damping rate, respectively. In equation (7), *α*(*V*) is defined as the voltage tunable spatial decay rate that depends on the doping value and the applied bias voltage. Moreover, *S* = *ε*_*s*_ − *ε*_∞,*pGaAs*_ is the oscillator strength using Lyddane-Sachs-Teller relation (i.e. 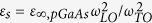) where *ω*_*LO*_ denotes the longitudinal optical (LO) phonon resonance frequency^14^,^15^. Since the average carrier density is tunable with the applied voltage, the plasma frequency at each voltage can directly be achieved using 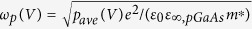 where *e* is the electron charge amount, *m*^∗^ is the effective mass and can be computed using heavy-hole *m*_*hh*_ and light-hole *m*_*lh*_ masses^8^,^9^,^10^,^35^. In the current work, the doping density is taken as *p* = 9.5 × 10^16^ cm^−3^, a somewhat moderate density, and the corresponding values for computing *ε*_*pGaAs*_(*ω*, *z*, *V*) used as given in Table 2. According to Fig. 1d and its relevant inset the maximum density of carriers at the interface and also the spatial decay which are bias voltage dependent functions can be approximated by 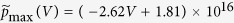, and *α*(*V*) = (*V* + 2) × 10^7^, respectively. It should be noted that in equation (7) we considered the values of 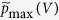 being normalized to the maximum density of carriers at V = +1 V.

**Figure 2:**
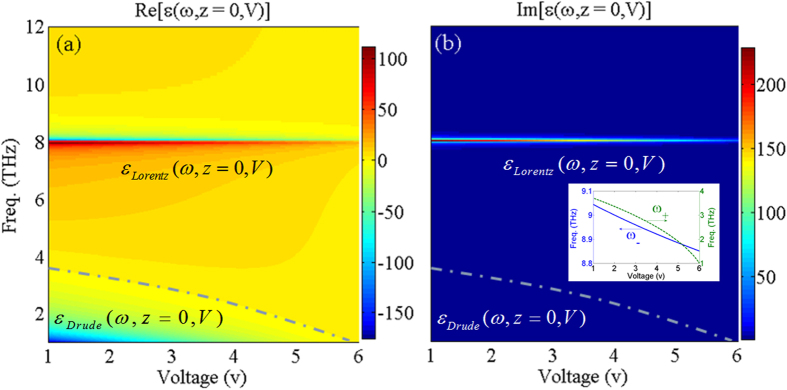
The frequency and bias voltage behavior of (**a**) real and (**b**) imaginary parts of the dielectric function of *ε*_*pGaAs*_(*ω*, *z*, *V*) using equation (7). The inset shows the behavior of ENZ frequencies at *ω*_±_ versus bias voltage.

Figure 2 shows the frequency and bias voltage dependency of the real (Fig. 2a) and imaginary (Fig. 2b) parts of *ε*_*pGaAs*_(*ω*, *z*, *V*), obtained from equation (7), at *z* = 0 (i.e. the interface). As can be seen from Fig. 2a,b the frequency dependency of the dielectric permittivity function for the moderately *p*-doped GaAs medium can be separated into two regions: (1) The Drude model part which is due to the effect of free carriers, and (2) The Lorentz model part due to the vibration of phonons in the semiconductor medium which causes asymptotic points occurring around *ω*_*TO*_.

Figure 2a,b indicate that by increasing the bias voltage, the amplitude of the real and imaginary parts of *ε*_*pGaAs*_(*ω*, *z*, *V*) are decreasing gradually due to less contribution of free carriers at higher bias voltages. By increasing the voltage, both maximum and average density of carriers (See cubes in Fig. 1d) decrease near the interface in an exponential fashion [i.e., *α*(*V*)], (See the circles in Fig. 1d). The latter property is a result of the band bending that does not favor hole accumulation near the interface.

As seen in Fig. 2(a), the real part of the *ε*_*pGaAs*_(*ω*, *z*, *V*) may take negative, zero, and positive values and thus each photonic device fabricated based on moderately *p*GaAs may operate with negative, zero, and positive permittivity. Furthermore it has been shown that one of the main emerging areas in designing integrated photonic and terahertz devices is the epsilon-near-zero (ENZ) materials in which, as the frequency tends to zero, the wave-front exhibits negligible spatial variations^37^,^38^. Thus, when investigating the optical properties of materials, one of the interesting areas is theoretically and/or experimentally studying the situations in which the ENZ condition may occur. Unlike noble metals, and due to the interaction effects between free carriers and lattice phonon resonances in the *p*GaAs medium, the ENZ behavior occurs at frequencies different from *ω*_*p*_(*V*). Considering 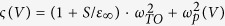, it can be shown that the zero values of the permittivity function may occur at 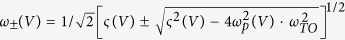. The inset of Fig. 2b shows the frequency and voltage variations of *ω*_+_ (dashed curve), and *ω*_−_ (solid curve), respectively. The results suggest that increasing the bias voltage leads to a red-shift of both resonant frequencies, whereas, the plasma frequency is much lower than the transverse phonon resonance. For frequency region of *ω*_*TO*_ to *ω*_*LO*_, the real part of permittivity is negative so that for small frequency variations (Δ → 0) around *ω*_*TO*_, 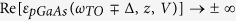. Due to the TO phonon damping constant, the asymptotic values of the dielectric function are finite and the negative values of permittivity for the *p*GaAs medium can be achieved at frequencies lower than *ω*_*p*_ and frequencies slightly higher than *ω*_*TO*_.

It is well-known that plasmons are collective oscillation of free electrons in a conducting (or a semiconducting) medium. This inhomogeneous nature of carrier distribution formally introduces a position dependent conductivity to the system. This position wise varying conductivity introduces additional terms into the otherwise well-known plasmonic mode analysis, leading to the possibility of the interface supporting the resonances reported here. In the following section, we will theoretically derive the dispersion relation of the DE-*p*GaAs interface considering the inhomogeneously distributed carriers and discuss the surface modes excited at the interface.

## Solution of Surface Plasmon and Phonon Polaritons At the Biased DE-*p*GaAs Interface

To investigate the plasmon excitation at the DE-*p*GaAs interface, we obtain the plasmon dispersion relation using the solution of wave equation with inhomogeneous material properties, as the charge density in the SC medium changes due to the significant penetration of the electric field inside the SC medium. Due to the spatial inhomogenity of the carrier distribiution in the biased *p*GaAs medium, the wave equation should be solved for the interface of DE-*p*GaAs as an inhomogenous media. Considering the dielectric function of the moderately *p*-doped GaAs as *ε*_*pGaAs*_(*ω*, *z*, *V*) [See equation (7)] and illuminating an incident transverse magnetic (TM) field at the interface, the following relation for the wave equation is obtained^39^:


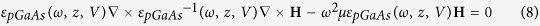


In equation (8), *β*(*V*) is the wave vector component parallel to the interface (i.e., along *x*-axis). For *z* > 0 region, equation (8) yields:


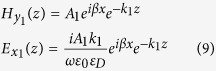


and for *z* < 0:


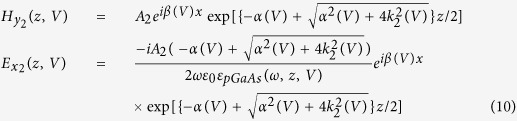


Using the continuity of tangential magnetic and normal electric fields at the boundary of *z* = 0 and considering *χ*(*V*) = *ε*_*pGaAs*_(*ω*, *z*, *V*)/*ε*_*D*_ we obtain:


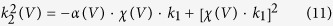


The perpendicular component of the wave vector (i.e. *k*_1_ and *k*_2_(*V*)) is given as:


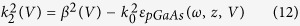



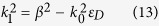


By substituting equations (12) and (13) into equation (11), the dispersion relation at the interface can be obtained as follows:


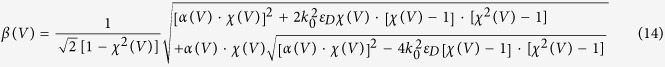


Equation (14) provides the dispersion relation at the interface of the DE and the moderately *p*-doped GaAs mediums. As discussed earlier, due to the significant penetration of the electric field inside the semiconductor and the solution of Poisson’s equation, the carrier distribution near the interface is not uniform, but follows an exponential decay starting from the interface (under bias). Since there are at least two orders of magnitude between the dielectric relaxation time of the semiconductor (at the order of nanoseconds) and the optical frequency timescale, the carrier distribution may not be altered by the incoming radiation. Moreover the phonon decay rate, which is much slower than the period of the optical radiation, is not expected to influence the carrier distribution at the semiconductor-dielectric interface. As it is shown in the following discussions and results, the optical properties and asympthotic resonances of the dispersion curve of the surface waves obtained from equation (14) can be tuned through the applied voltage.

A major difference between the dispersion relation of a homogeneous medium (typically encountered in metal plasmonics) and inhomogeneous medium, is about the spatial decay rate (i.e., *α*(*V*)) which leads to a voltage dependent term in 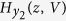 and 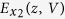 fields. As the *α*(*V*) term equals to zero for homogeneous materials, we verified the results of the calculations given in equation (14) for the limiting case of DE-metal interfaces. We used silver and gold as the metals, where the spatial variations of their dielectric function at the interface reduces to a simple step function. Under such considerations, our results in equation (14) simplifies to the well-known dispersion relations of the DE-metal interfaces.

As mentioned previously, for the *p*-doped GaAs medium it is expected that for *ω* < *ω*_+_(*V*) and also *ω*_*TO*_ < *ω* < *ω*_−_(*V*) the negative real permittivity can be achieved. To get a better understanding of the free carriers and lattice phonon resonance effects on excitation of surface waves at the DE-*p*GaAs interface, the asymptotic behavior of the excited surface waves due to each part is investigated. For the lower doping values, the negative permittivity occurs at lower frequencies due to lower plasma frequency. Thus, weaker interaction can occur between free carrier’s motion (at lower frequencies) and lattice phonon resonances (at much higher frequencies). Therefore, the frequency and voltage variations of the dielectric function are dominated by the Drude part of equation (7) at lower frequencies. In order to understand the effect of SPPs and SPhPs in the asymptotic behavior of the dispersion relation, we separately considered the impact of the Drude and Lorentz parts on the spectral results of equation (14) using doping data values and the voltage dependent decay rate shown in Fig. 1d and the inset.

It can be shown that considering *ε*_*pGaAs*_(*ω*, *z*, *V*) = *ε*_*Drude*_(*ω*, *V*). *P*(*z*, *V*), the dispersion diagram consists two surface plasmon resonant asymptotic frequencies at:


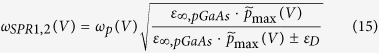


**Figure 3:**
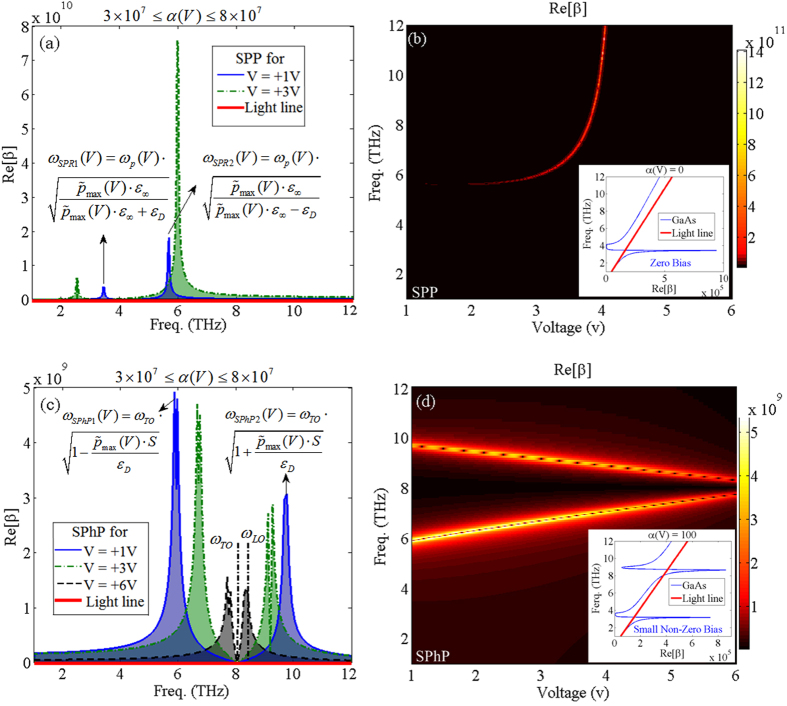
(**a,c**) The spectrum of dispersion curve of free carriers (Drude model), and lattice phonon resonances (Lorentz model), respectively, using equation (14) for V = +1 V (solid-line), V = +3 V (dashed-dotted-line), and V = +6 V (dashed-line), compared to light line (thick-solid-line) under *α*(*V*) ≠ 0 condition. (**b,d**) The two dimensional variations of the free carriers and also lattice phonon resonances dispersion curve versus changing voltage and frequency, correspondingly. The insets demonstrate the dispersion curve of zero, and small none-zero bias voltage applied to the structure [i.e., *α*(*V*) = 0, and *α*(*V*) = 10^2^] using the Drude model in equation (14).

These asymptotic frequency values are voltage tunable through changing the carrier density at the boundary of the inhomogeneous medium. It should be noted that although variations of *ε*_*D*_ leads to the change of the electrical potential between the DE and the doped GaAs medium which in turn results in variations of the maximum and average density of free carriers, the investigation of different values of the DE medium permittivity is not the main focus of this article and the permittivity data presented in Table 2 are used for these calculations (i.e. *ε*_*D*_ = 5). Figure 3a,c demonstrate the dispersion curve due to the free carriers and the lattice phonon vibrations, based on equation (14) for V = +1 V (solid-line), +3 V (dashed-dotted-line), and +6 V (dashed-line), compared to light line (thick-solid-line), respectively. Figure 3b,d depict the variations of the free carriers and also lattice phonon resonances dispersion relation versus changing voltage and frequency, respectively. It should be noted that the asymptotic resonance frequencies for V = +6 V occur out of the considered frequency band, thus, the curve related to this voltage is not depicted in Fig. 3a. Physically, as the average density of carriers is decreased, the strength of the collisions between free electrons is reduced so that this mechanism leads to weaker interaction between the free carriers. Hence, according to Fig. 3a,b, by increasing the bias voltage *ω*_*SPR*1_(*V*), and *ω*_*SPR*2_(*V*) are red-shifted and blue-shifted, respectively. The latter phenomenon is due to decreasing the average density of carriers [directly affects the *ω*_*P*_(*V*) frequency] and maximum carrier density at the interface by increasing the bias voltage. As mentioned earlier, in order to confirm the results obtained from equation (14), we set *α*(*V*) = 0 which is identical to homogenously distributed carriers. The dispersion curve for zero bias voltage is depicted in the inset of Fig. 3b which demonstrates similar plasmonic properties of noble metals but in a lower frequency band. Slightly increasing the bias voltage [i.e., a small none-zero bias]; leads to small values of spatial decay rate, i.e. *α*(*V*). Considering, for instance, *α*(*V*) = 10^2^ results in two asymptotic resonant frequencies in the dispersion curve which are double frequency poles of equation (14). The inset of Fig. 3d shows the dispersion curve at the interface for small none-zero applied voltage with *α*(*V*) = 10^2^.

We also investigated the optical behavior at the interface when the dielectric function of the doped region is dominated by the Lorentz part of equation (7), which yields the optical phonon vibrations of the SC lattice. Two resonant asymptotic frequency peaks may occur around *ω*_*TO*_, which can be obtained theoretically through 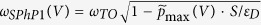 and 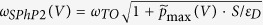, respectively. According to Fig. 3c, the asymptotic resonant frequency values due to the lattice vibrations can be tuned via the bias voltage. As the bias voltage is increased, *ω*_*SPhP*1_ blue-shifts and *ω*_*SPhP*2_ experiences a red-shift. The closed form formulations of *ω*_*SPhP*1_ and *ω*_*SPhP*2_ shows that the important parameter in changing the resonant frequencies is the maximum carrier density at the interface which can be reduced by increasing the applied voltage.

**Figure 4:**
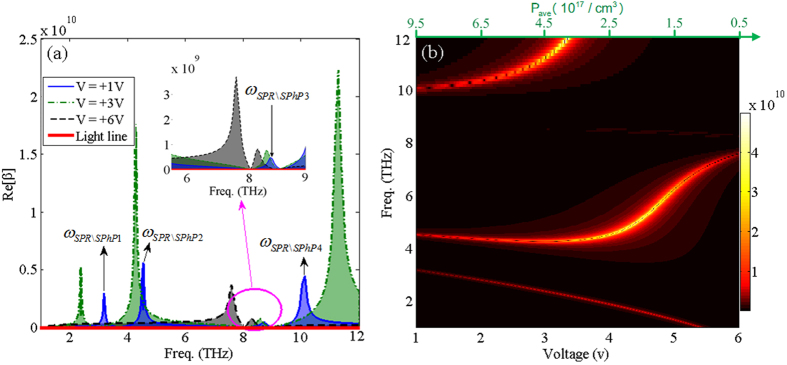
(**a**) The dispersion curve of the DE-pGaAs interface using equation (14) under bias voltage of V = +1 V (solid-line), +3 V (dashed-dotted-line), and +6 V (dashed-line) compared to the light line (thick-solid-line) with 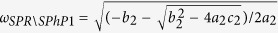, 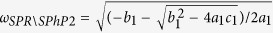, 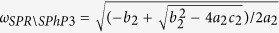, and 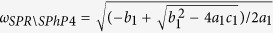. (**b**) The variations of the dispersion curve versus changing voltage and frequency. The upper horizontal axis indicates the corresponding average carrier density.

The *p*GaAs medium naturally includes both the free carriers accumulation and also optical phonon resonances via the spatially varying Drude-Lorentz dielectric function of *p*GaAs in the terahertz regime. To investigate the optical properties of the DE-*p*GaAs it is necessary to consider the *p*GaAs medium taking into account both with *ε*_*Drude*_(*ω*, *V*) and also *ε*_*Lorentz*_(*ω*) in the dispersion relation and the discussions that will follow. Considering both parts in Fig. 4, we study the overall impact of inhomogeneously distributed free carriers and phonon vibration interactions on the excited surface waves at the junction of DE and *p*GaAs mediums. Figure 4a demonstrates the dispersion curve for V = +1 V (solid-line), +3 V (dashed-dotted-line), +6 V (dashed-line), and light line (solid-line), correspondingly. Furthermore, Fig. 4b elucidates the variations of the dispersion curve versus different values of frequency and bias voltages. By setting the denominator of equation (14) equal to zero, four asymptotic resonant frequencies can be obtained for each relevant voltage in Fig. 4 as:


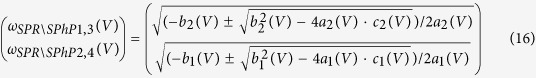


where *a*_1,2_(*V*) = 1 ± 1/*e*(*V*), 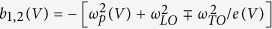, 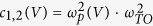, and 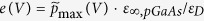. According to equation (16), both the average and maximum density of free carriers at the boundary strongly affect the asymptotic resonant peak frequencies and also the resonant strength of the dispersion spectrum.

Based on the results shown in Fig. 4a,b, however, for applied voltages less than +3 V *ω*_*SPR*\*SPhP*1_(*V*), *ω*_*SPR*\*SPhP*2_(*V*) and *ω*_*SPR*\*SPhP*3_(*V*) are red-shifted and *ω*_*SPR*\*SPhP*4_(*V*) is blue-shifted by increasing the bias voltage; while, for voltage values higher than +4 V, *ω*_*SPR*\*SPhP*2_(*V*) is dramatically blue shifted. In fact, for the supposed junction of the DE and moderately doped GaAs mediums; since the carrier’s density is decreased by increasing the bias voltage in the inhomogeneous layer; the interaction of free carriers with the lattice surface phonon vibrations is weakened. The latter issue can be considered to play the main role in the interaction mechanism of the SPPs and SPhPs at the interface and also the spectral shifts of the asymptotic resonant peaks of the dispersion curve. The effect of varying bias voltage on the spectral shifts and also frequency band gaps between the asymptotic resonant values can be further investigated. This was achieved by increasing the voltage on each pair of Δ*ω*_*j*,*i*_(*V*) = *ω*_*SPR*\*SPhPj*_(*V*) − *ω*_*SPR*\*SPhPi*_(*V*) frequencies with *j* ≠ *i* and *j* = 2–4, and *i* = 1–3, respectively.

**Figure 5:**
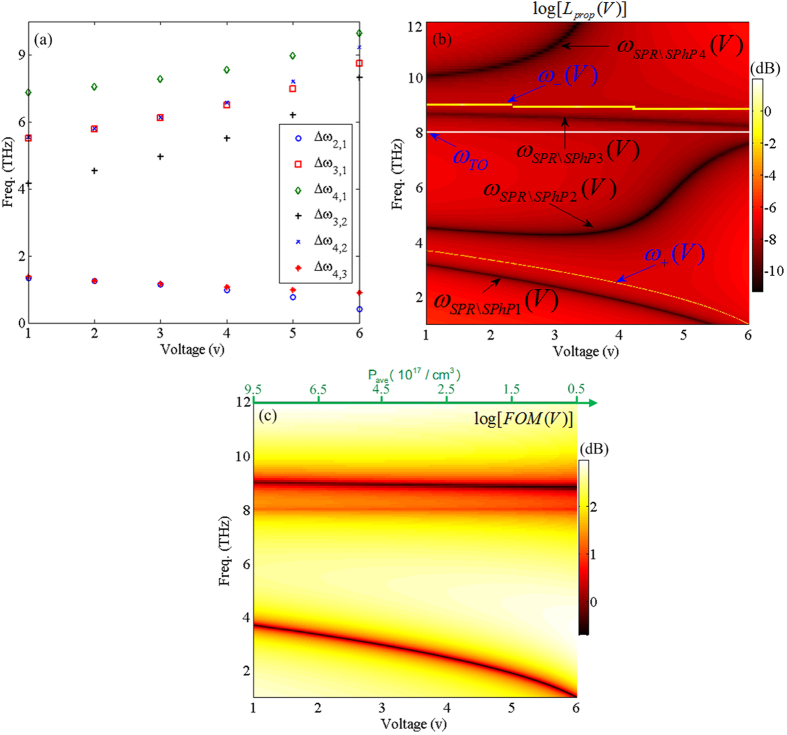
(**a**) Frequency band gaps between asymptotic resonant frequencies versus bias voltage for Δ*ω*_2,1_ (circles), Δ*ω*_3,1_ (cubes), Δ*ω*_4,1_ (diamonds), Δ*ω*_3,2_ (pluses), Δ*ω*_4,2_ (crosses), and Δ*ω*_4,3_ (stars), respectively. (**b**) The spectrum variation of the propagation length in logarithmic scale at the DE-*p*SC interface versus changing bias voltage. (**c**) Figure of merit for propagating polaritons (See ref. 17).

Figure 5a shows the variations of frequency band gaps between Δ*ω*_2,1_ (circles), Δ*ω*_3,1_ (cubes), Δ*ω*_4,1_ (diamonds), Δ*ω*_3,2_ (pluses), Δ*ω*_4,2_ (crosses), and Δ*ω*_4,3_ (stars) versus tuning the applied voltage. Figure 5a suggests that except for Δ*ω*_3,2_, the asymptotic frequency gaps are decreased by increasing the voltage which is due to reduction of carriers density at the interface.

Another finding related to the system in this work is the propagation length of the surface waves. Since the wavenumber of the surface waves can be tuned through the applied bias voltage, the corresponding propagation length of these waves along *x*-axis can be tuned using *L*_*prop*_(*V*) = 1/(2 Im[*β*(*V*)]). Figure 5b illustrates the spectrum of the propagation length in terms of decibels (dB) at *z* = 0 boundary versus changing bias voltage in far-IR regime. Since the propagation length has strong dependency on the applied voltage, different propagation lengths between several nanometers up to near millimeters frequencies can be supported by the structures investigated here. Therefore by selecting the bias value and the associated frequency, longer propagation lengths can be achieved, which is desired in most plasmonic and phononic applications. Based on our results in Fig. 5b, for frequencies between 1 THz < Freq < 2 THz and +1 V < voltage < +2 V, a maximum propagation length of around 1.2 mm can be achieved. In addition, for 10 THz < Freq < 12 THz and +5 V < voltage < +6 V, a propagation length of 0.8 mm, and also for 2 THz < Freq < 4 THz regime keeping the bias on the system as +5 V < voltage < +6 V the propagation length can varied from around 0.5 mm to around 0.8 mm, respectively. We determined, based on our calculations, that the aforementioned propagation lengths are achieved due to the strong coupling of the plasmonic and phononic polaritons as shown in Fig. 5b. In Fig. 5c, we plot the figure of merit (FOM) [17] that is a measure of the propagation length and confinement of the surface modes at the interface. According to Fig. 5c, it can be seen that the maximum FOM occurs at frequencies greater than *ω*_−_(*V*) as well as frequencies between *ω*_+_(*V*) to *ω*_*TO*_ and those lower than *ω*_*SPR*\*SPhP*1_(*V*) which is about 2.5 dB (where maximum propagation length can be obtained). Note that, for these frequency regions, the FOM is about an order of magnitude more enhanced than values reported for intrinsic GaAs [17] due to the doping levels considered in our study. In Fig. 5c, high FOM regions corresponds negative values of the real part of the dielectric function. It is obvious that for the average carrier distribution higher than 4.5 × 10^17^ cm^−3^ maximum FOM occurs for *ω*_*SPR*\*SPhP*1_(*V*), and for carrier distributions lower than 3 × 10^17^ cm^−3^ it occurs for frequencies between *ω*_+_(*V*) to *ω*_*TO*_.

## Conclusions

In this study, we showed that the plasmon dispersion curve can be engineered through an external bias using the inherent properties of the DE-SC interface. The main finding of this work is that the carrier distributions on a moderately *p*-doped GaAs layer interfacing a dielectric can be tailored using an external bias, allowing the tuning of SPPs and SPhPs interaction in the far IR regime. Increasing the bias voltage leads to a reduced density of carriers at the interface, which results in lower plasma frequencies at the DE-SC interface while a lower bias (around 1 V) generates an opposite trend. Our findings indicate a strong coupling of free carriers and optical phonon vibrations. We computed four asymptotic resonant frequencies, some of which indicate the possibility of exciting surface waves at far IR frequencies. The strong coupling of surface plasmons and surface phonons increased the propagation length of the surface waves spanning a spectrum starting from the far-IR regime all the way to almost millimeter wavelengths. The FOM for the system considered here is about an order of magnitude more enhanced than values reported for intrinsic GaAs due to the doping levels considered in this work. Our findings are significant for the design of waveguides and optics based on exploiting the inherent properties of semiconductors.

## Additional Information

**How to cite this article**: Janipour, M. *et al*. Tunable Surface Plasmon and Phonon Polariton Interactions for Moderately Doped Semiconductor Surfaces. *Sci. Rep.* **6**, 34071; doi: 10.1038/srep34071 (2016).

## Figures and Tables

**Table 1:** Parameters used for calculate the carrier density at the DE-*p*GaAs interface using equations (1)–(5) .

*ε*_*DC*,*pGaAs*_	*E*_*F*_(eV)	*N*_*C*_(cm^−3 ^E^−1^)	*N*_*V*_(cm^−3 ^E^−1^)	*N*_*A*_(cm^−3^)	*E*_*A*_, *E*_*D*_(eV)	*E*_*C*_, *E*_*V*_(eV)
12.9	−5.2	10^19^	10^19^	10^17^, 10^18^	−0.05	−4.05, −5.15

**Table 2:** Data parameters used to calculate the permittivey function of the *p*GaAs medium.

*ε*_∞,*pGaAs*_	*ε*_*D*_	*ω*_*TO*_(THz)	*ω*_*LO*_(THz)	Γ(THz)	*γ*(THz)
10.89	5	8	8.4	0.055	0.01
